# New allele of *C. elegans* gene *pign-1*, named as *xyz11*

**DOI:** 10.17912/micropub.biology.000088

**Published:** 2019-01-18

**Authors:** Tetsuya Narimatsu, Shinji Ihara

**Affiliations:** 1 Department of Chemical and Biological Engineering, Ariake National College of Technology, 150 Higashihagio-machi, Omuta, Fukuoka 836-8585, Japan.

**Figure 1.**
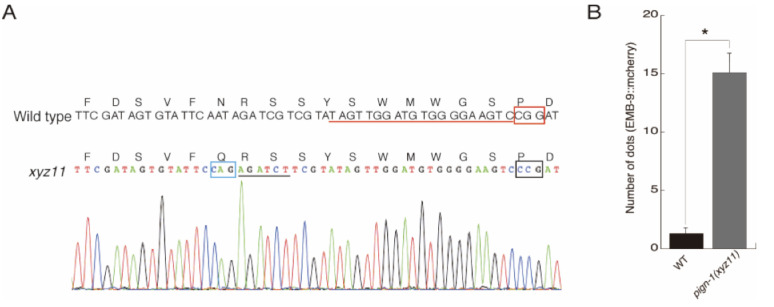
A. Sequence confirmation of the *pign-1(xyz11)* allele. Nucleotides and corresponding amino acid sequences in the wild-type *pign-1* (upper) and *xyz11* (bottom) are shown. The single guide RNA (sgRNA) target sequences are underlined in red, and the protospacer adjacent motif (PAM) is indicated by a red box. We used a single strand oligo, a homologous repair template that contains a missense mutation of AAT to CAG (blue box, corresponding to the N127Q mutation) together with the disrupted PAM motif (black box) that prevents re-digestion after homologous repair. We also introduced XhoII recognition site (black line) without altering the corresponding amino acid sequence in the single strand oligo. The XhoII site was used to identify the targeted mutant. B. Quantification of type IV collagen:mCherry (EMB-9::mCherry) aggregates in body wall muscle cells (n ≥ 10 worms at L4 stage). Asterisks denote statistically significant differences (P<0.01; Student’s t-test). Error bars represent the standard error of the mean (± s.e.m.).

## Description

The *C. elegan*s ortholog of PIGN (phosphatidylinositol glycan anchor biosynthesis, class N) is encoded by *pign-1.* PIGN-1/PIGN is known to be an enzyme that catalyzes the transfer of phosphoethanolamine (EtNP) to the first mannose residue at precursors of glycosylphosphatidylinositol (GPI) anchor (canonical function) and the enzymatic activity is essential for the viability of *C. elegans* (Gaynor et al., 1999; Hong et al., 1999; Ihara et al., 2017). We recently identified a non-canonical function of PIGN-1 to prevent protein aggregation within the endoplasmic reticulum (ER) independently of its function in GPI biosynthesis (Ihara et al., 2017).

Although *C. elegan*s *pign-1* contains four potential N-glycosylation sites (N-X-S/T), the N127 (human N128) in the first loop at the ER side of PIGN-1 is only evolutionally conserved in yeast, worm, and vertebrate genomes (Gaynor et al., 1999). We examined GlycoProtDB which is a glycoprotein database providing information of Asn (*N*)-glycosylated proteins and confirmed really *N*-glycosylated at the asparagine 127 of PIGN-1 in *C. elegans*, GlycoProtDB (CELE_Y54E10BR.1) https://acgg.asia/db/gpdb/GPDB0000140. Using CRISPR/Cas9 genome engineering, we replaced asparagine 127 (human N128) in potential *N*-glycosylation site in the first loop at ER side of PIGN-1 with glutamine, which cannot be *N*-glycosylated (Figure 1A).

The *pign-1(xyz11:N127Q)* animal were measured for protein aggregation within the ER using EMB-9::mCherry (*qyIs44*), which is quantitatively measures the secretion efficiency from ER. Compared to wild-type animals, the *pign-1(xyz11:N127Q)* mutant showed a statistically significant protein aggregation in the body wall muscle cells (Figure 1B). In addition, *pign-1(xyz11:N127Q)* mutant was viable and fertile, suggesting that the EtNP transfer activity to GPI anchor was not disrupted.

## Reagents

The *qyIs44* [emb-9p::EMB-9::mCherry] was used for the injection strain. Animals were raised at 20 °C. The sgRNA vectors were microinjected together with 50 ng/ul single strand oligo, 50 ng/ul pDD162, and 50 ng/ul sur-5::gfp in *qyIs44* animals. We selected candidate mutants based on abnormal localizations of EMB-9::mCherry, and confirmed the mutations using PCR.

The sgRNA plasmid was derived from Addgene plasmid 46169.

pDD162 (Peft-3::Cas9 + Empty sgRNA) vector from Bob Goldstein (Addgene plasmid # 47549 ; http://n2t.net/addgene:47549 ; RRID:Addgene_47549) was used for Cas9 expression (Dickson et al., 2013).

Single strand oligo used to generate *xyz11*: CCAGTTCAATTCGATAGTGTATTCCAGAGATCtTCGTATAGTTGGATGTGGGGAAGTCCCGATATTGTGAACCTTTTCGATGATCT. Strain: IHR-177 *pign-1(xyz11)*I. It will be sent to the CGC.
